# The Electronic Transport Channel Protection and Tuning in Real Space to Boost the Thermoelectric Performance of Mg_3+*δ*_Sb_2-*y*_Bi*_y_* near Room Temperature

**DOI:** 10.34133/2020/1672051

**Published:** 2020-02-28

**Authors:** Zhijia Han, Zhigang Gui, Y. B. Zhu, Peng Qin, Bo-Ping Zhang, Wenqing Zhang, Li Huang, Weishu Liu

**Affiliations:** ^1^Department of Materials Science and Engineering, Southern University of Science and Technology, Shenzhen 518055, China; ^2^School of Materials Science and Engineering, University of Science and Technology Beijing, Beijing 10083, China; ^3^Department of Physics, Southern University of Science and Technology, Shenzhen 518055, China; ^4^Academy for Advanced Interdisciplinary Studies, Southern University of Science and Technology, Shenzhen 518055, China; ^5^Shenzhen Engineering Research Center for Novel Electronic Information Materials and Devices, Southern University of Science and Technology, Shenzhen 518055, China

## Abstract

The optimization of thermoelectric materials involves the decoupling of the transport of electrons and phonons. In this work, an increased Mg_1_-Mg_2_ distance, together with the carrier conduction network protection, has been shown as an effective strategy to increase the weighted mobility (*U* = *μm*^∗3/2^) and hence thermoelectric power factor of Mg_3+*δ*_Sb_2-*y*_Bi*_y_* family near room temperature. Mg_3+*δ*_Sb_0.5_Bi_1.5_ has a high carrier mobility of 247 cm^2^ V^−1^ s^−1^ and a record power factor of 3470 *μ*W m^−1^ K^−2^ at room temperature. Considering both efficiency and power density, Mg_3+*δ*_Sb_1.0_Bi_1.0_ with a high average ZT of 1.13 and an average power factor of 3184 *μ*W m^−1^ K^−2^ in the temperature range of 50-250°C would be a strong candidate to replace the conventional n-type thermoelectric material Bi_2_Te_2.7_Se_0.3_. The protection of the transport channel through Mg sublattice means alloying on Sb sublattice has little effect on electron while it significantly reduces phonon thermal conductivity, providing us an approach to decouple electron and phonon transport for better thermoelectric materials.

## 1. Introduction

Thermoelectric (TE) materials offer the convenience to convert the widely distributed waste heat into electric power directly, which is highly desired for the autonomous operation of the Internet of things (IoT) in recent years. The conventional room temperature (RT) thermoelectric material, Bi_2_Te_3_ family, dominates the market of the solid-state refrigeration [1, 2]. However, its mediocre mechanical property and the extremely low abundance of Te element limit its application [3]. The past years have witnessed great progress in developing medium-temperature thermoelectric materials, but not so much in near room temperature TE materials. So far, there is no candidate material that can compete with the Bi_2_(Te,Se)_3_ family in terms of near room temperature TE performance. In our previous report, we have shown that the Mn-doped Mg_3+*δ*_Sb_1.5_Bi_0.5_ ((ZT)_avg_ = 1.05, *K*_IC_ = 2.2‐3.0 MPa m^1/2^) would be a very promising candidate for substituting the Bi_2_Te_3−*x*_Se*_x_* family ((ZT)_avg_ = 0.9–1.0, *K*_IC_ = 0.6‐1.3 MPa m^1/2^) in the temperature range of 50–250°C because of the comparable average ZT and much higher fracture toughness [4]. It is noted that intensive efforts have been made into searching high ZT composition in the Bi-rich Mg_3+*δ*_Sb_2-*y*_Bi*_y_* with varying doping [5–7]. Imasato et al. researched Bi content-dependent thermoelectric properties and discovered that Mg_3+*δ*_Sb_0.6_Bi_1.4_ shows exceptional thermoelectric performance [8]. Ren et al. produced a thermoelectric cooling couple with Mg_3+*δ*_Sb_0.5_Bi_1.5_ and Bi_0.5_Sb_1.5_Te_3_, which realized Δ*T* around 90 K at the hot-side *T* of 350 K. Further improvement on the power factor is desirable for power generation applications [9]. However, the power factor of the reported Mg_3+*δ*_Sb_1.5_Bi_0.5_ is still lower than that of the Bi_2_Te_3−*x*_Se*_x_* family, which could lead to a reduced power density of the thermoelectric power generator. Further enhancement in the PF of Mg_3+*δ*_Sb_1.5_Bi_0.5_ is thus much desired.

However, it is challenging to decouple the transport of electrons while tuning the thermoelectric properties [10]. Electron-phonon interaction and defects would affect electron and phonon transport at the same time. The material parameter *B* (*B* ∝ *μm*^∗3/2^/*κ*_lat_) is commonly used as a guideline to boost thermoelectric performance of materials, where *μ* and *m*^∗^ are the carrier mobility and effective mass, respectively [11]. Usually, a material that has multicarrier valleys and small DOS effective mass for each valley could have a large power factor (*S*^2^*σ*) [12], and a material with heavy elements can have low intrinsic *κ* [13]. For instance, Bi_2_Te_3_ and PbTe are known as conventional thermoelectric materials. Also, alloying has been greatly used to reduce the lattice thermal conductivity after Ioffe's [14] and Goldsmid's [15] pioneering works. However, the decrease in *κ* from alloying is usually offset by the reduction in *μ* from alloying [16]. Recently, Liu et al. have found that the small atomic size difference between the period 5 elements (Zr, Nb) and period 6 elements (Hf, Ta) moderates alloying's negative impact on carrier mobility in the half-Heusler system [17].

In 2016, Tamaki et al. reported an n-type Mg_3+*δ*_Sb_2_-based laminar Zintl compound with a high ZT of 1.5 at 442°C [18], which has attracted much attention in the thermoelectric community to enhance the peak ZT of Mg_3+*δ*_Sb_2_-based TE materials [19–22]. The most surprising feature of this material family is that the alloy disorder at anionic sites has little effect on carrier mobility. Favorable alloying sites are known in the Bi_2_Te_3_ family, i.e., n-type Bi_2_Te_3_ has a favorable site at the Te sublattice while the p-type one has a favorable site at the Bi sublattice. Similar favorable doping sites have been observed in the PbTe [23, 24] and Mg_2_Sn [25] systems. Wang et al. suggested that the favorable dopants should be on the site that is of less influence on the charge-conducting band [24]. Recently, Yang et al. proposed a conduction network in the real space: the vast majority of atoms formed a conductive framework for charge carriers, while the chief role of the remaining atoms was to scatter phonons [26]. If the alloying elements or dopants get into the sublattice away from the conduction network, they would have little impact on carriers' transport. Tamaki et al. also pointed out the conduction network, formed by the 3s-like orbitals of Mg^2+^ and the weakly hybridized atomic orbitals of [Mg_2_Sb_2_]^2-^ in Mg_3+*δ*_Sb_2_-based layered Zintl compound, but not connecting with the feature that alloying has little effect on carrier mobility. We believe that this carrier conductive network forms a favorable carrier transport channel in real space. It was found that the disordering Bi/Sb at the anionic site is “far away” from the electronic transport channel, which explains the fact that the electrical conductivity of Mg_3+*δ*_Sb_1.5_Bi_0.5_ is very sensitive to the Mg vacancy but not to the disordering Bi/Sb [18, 27, 28]. Experimentally, excess Mg is necessary to suppress the formation of Mg vacancy and obtain stable n-type samples [19]. However, as suggested by Tamaki et al., Mg vacancy is still a favorable intrinsic defect even in a Mg-rich condition. In our previous work [4], we have shown that the Mn dopant at Mg_4_-tetrahedron interstitial site provides an attracting force to suppress the formation of the Mg vacancy.

In this work, we will theoretically show a more unique character of the carrier conduction network: by increasing the Mg_1_-Mg_2_ distance, we can increase the weighted mobility *U* and enhance the power factor (*S*^2^*σ*) through the increased carrier mobility. Experimentally, we found the Bi-rich Mg_3+*δ*_Sb_2-*y*_Bi*_y_* with interstitial dopant Mn and anionic dopant Te has a recorded high carrier mobility of 247 cm^2^ V^−1^ s^−1^ and a high power factor of 3470 *μ*W m^−1^ K^−2^ at room temperature in the Mg_3+*δ*_Sb_0.5_Bi_1.5_ sample. The Mg_3+*δ*_Sb_1.0_Bi_1.0_ possessed a high average ZT of 1.13 and an average PF of 3184 *μ*W m^−1^ K^−2^ in the temperature range of 50-250°C.

## 2. Results and Discussion

**Figure 1:**
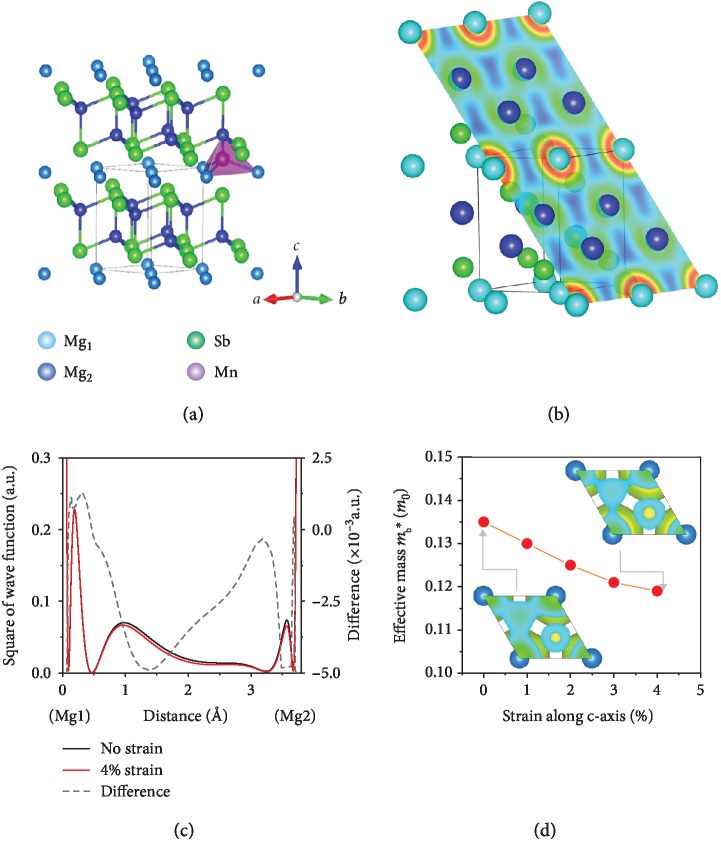
(a) Crystalline structure of Mg_3_Sb_2_ with Mn at the interstitial site. (b) Charge density in (011) plane. (c) The square of real-space wave function (i.e., the “charge” density) at CBM along Mg_1_-Mg_2_ line for Mg_3_Sb_2_ bulk (black solid line), under 4% strain (red solid line) along the *c*-axis and the difference (black dotted line) between “charge” density of Mg_3_Sb_2_ under 4% strain and bulk. Clear reduction of the covalence-like overlap of Mg_1_ and Mg_2_ 3s orbitals between Mg_1_ and Mg_2_ is shown with the strain and the released “charge” mostly goes to Mg_1_. (d) Strain-dependent effective mass at conduction band edge. The inset compares the calculated charge density distribution of the two cases in the a-b cross section at the *c*-axis fractional coordinate *z* = 0.18, with the same setting of isosurface around 15% of the maximum values.

Figure 1 shows the effect of increased Mg_1_-Mg_2_ distance in real space on the band structure and Hall mobility. Our motivation was to find a way to further tailor the charge conductive network in real space and make it more favorable for the transport of electrons. Mg_3_Sb_2_ has a trigonal structure (space group: $P\bar{3}m1$) and a layered structure with alternate layers of Mg and [Mg_2_Sb_2_] in the ab-plane [18], as shown in Figure 1(a). Recently, Sun et al. suggested that part of the conduction band minimum (CBM) originates from the covalence-like bonding state of Mg_1_ 3s orbital and Mg_2_ 3s orbital and there was also a small amount of antibonding-like interaction between Mg_1_ 3s and Sb 5s orbitals, where Mg_1_ represents the Mg at the Sb-octahedral center (0, 0, 0) while Mg_2_ is the Mg at the tetrahedral center (0.3333, 0.6667, 0.3718(4)) [29]. Figure 1(b) shows the charge density in (011) plane of the trigonal Mg_3_Sb_2_. Detailed analysis of the band composition near CBM shows that the states near CBM consist of Mg_1_ 3s orbitals, Mg_2_ 3s orbitals, and Sb 5s orbitals. The most weighted contribution to conductive network comes from the dispersive Mg_1_ 3s orbitals. Furthermore, our first-principles calculations also suggest that an increase of Mg_1_-Mg_2_ distance reduces the overlap of Mg_1_ 3s and Mg_2_ 3s orbitals that constitute the Mg_1_-Mg_2_ “bond.” As the distance between Mg_1_ and Mg_2_ increases with strain, the difference of the squared wave function, between pristine and the one with 4% strain, shows that (i) the overlap of Mg_1_ 3s and Mg_2_ 3s orbitals at CBM reduces and (ii) the released charge would go to Mg_1_ 3s orbitals and enhance the antibonding between Mg_1_ 3s orbitals that is the most pronounced constituent around CBM (see Figure 1(c)). Therefore, it leads to a more dispersive band (i.e., lighter band) (see Figure 1(d)). The red line with solid circles in Figure 1(d) plots the DOS effective mass of conduction band minimum of Mg_3_Sb_2_ as a function of the strain along the *c*-axis. An almost linear decline in the band effective mass is observed when the strain increases from 1% to 4%. When acoustic phonon scattering is considered as the dominant scattering mechanism, the maximum power factor is proportional to the ratio *N*_*v*_/*m*^∗^ (the derivation is given in SI A). A smaller effective mass (lighter band) corresponds to the higher carrier mobility and the increased power factor [27, 30, 31]. Li et al. also suggested an enhanced power factor of Mg_3_SbBi as a biaxial strain was used in their first-principles calculation [28].

**Figure 2:**
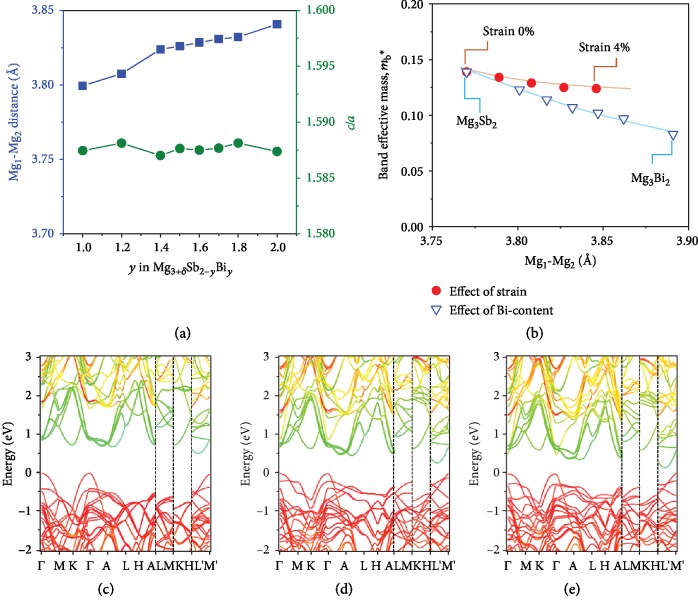
(a) Mg_1_-Mg_2_ distance and the ratio *c*/*a* as function of Bi content. (b) Band effective mass *m*_b_ as a function of the local distance of Mg_1_-Mg_2_ for the case with the *c*-axis strain and Bi alloying effect. (c–e) Calculated band structures for Mg_3_Sb_2-*y*_Bi*_y_* solid solutions with (c) *y* = 0.0, (d) *y* = 1.0, and (e) *y* = 1.5. In each figure, the color indicates the weight values (green: 1.0; red: 0.0) of Mg 3s orbitals in the band structures. The bands near CBM are mainly from Mg 3s orbitals.

Figure 2 provides more information on the relation between carrier effective mass of Mg_3_Sb_2_ and Mg_1_-Mg_2_ distance according to our first-principles calculations. Experimentally, partial substitution of Sb atoms with Bi atoms expands the crystalline lattice, as shown in Figure 2(a). The XRD patterns of Mg_3_Sb_2-y_Bi_y_ (*y* = 1.0~2.0) powders (grinded by SPS bulks) are given in Fig. [Supplementary-material supplementary-material-1] (SI). All the samples show a single phase with a La_2_O_3_-type trigonal structure. The lattice parameters and distance of Mg_1_-Mg_2_ are derived from the Rietveld refinement. An almost linear expansion is observed that the lattice parameter *c* varies from 7.296 Å to 7.416 Å (Fig. [Supplementary-material supplementary-material-1], SI) with increasing Bi content, indicating a complete solid solution of Mg_3_Sb_2_ and Mg_3_Bi_2_, and the *c*/*a* ratio remained around 1.587, which is close to that of pure Mg_3_Sb_2_. As a result, the Mg_1_-Mg_2_ distance increases from 3.832 Å to 3.891 Å as the Bi content increases from *y* = 1.0 to *y* = 2.0. The estimated Mg_1_-Mg_2_ distances in Mg_3_Sb_2_ and Mg_3_Sb_1.5_Bi_0.5_ are 3.761 Å and 3.786 Å, respectively. Figure 2(b) plots the effective mass for the single band (*m*_b_^∗^) as a function of Mg_1_-Mg_2_ distance. Here, both the *c*-axis strain and Bi alloying effect increase the Mg_1_-Mg_2_ distance. The DOS effective mass at the conduction band edge decreases with increasing Mg_1_-Mg_2_ distance. Bi alloying increases the distance between Mg_1_ and Mg_2_ and weakens the covalence-like bonding between Mg_1_ 3s and Mg_2_ 3s orbitals. However, the slopes of the two curves, as shown in Figure 2(b), are slightly different. The change in band effective mass mb^∗^ from Bi alloying is more complicated because (1) adding Bi into the system expands the lattice not only along the *c*-axis but also in the a-b plane (Fig. [Supplementary-material supplementary-material-1], SI) and (2) adding Bi leads to the upward shifting of valence bands and the narrowing of band gaps. The upward shifting of valence bands brings hybridization between Bi p orbitals from valence bands and Mg_1_ 3s orbitals from conduction bands. Such hybridization makes bands more dispersive, therefore lighter effective mass. A similar tensile strain-dependent reduction of band DOS effective mass was also reported in the typical semiconductor with well-known covalent bonds, such as Ge [32] and Si [33].

A closing band gap of Mg_3_Sb_2-y_Bi_y_ with increasing Bi content has been theoretically predicted by many researchers [27, 34]. Our previous experiments also proved this [4]. Zhang et al. suggested a transition from semiconductor to semimetal as the Bi content increases and goes higher than *y* = 1.5 (Mg_3_Sb_2-y_Bi_y_) [34]. However, in our calculation, the band gap remains as large as 0.17 eV for the sample Mg_3_Sb_0.5_Bi_1.5_ without spin-orbit correction. Our calculations for the Mg_3_Sb_2-y_Bi_y_ (*y* = 0, 0.5, 0.75, 1, 1.25, 1.5, and 2.0) family give the same trend as shown in Figures 2(c)–2(e) and Table 1. The narrowing of the band gap with increasing Bi concentration is attributed to the upward shift of valence band maximum since the valence band maximum mainly consists of Sb and Bi p orbitals, and Bi p orbitals are more dispersive and lie at higher energy levels than Sb p orbitals. The band shape at CBM does not change much within the calculated Sb/Bi ratio window, but the effective mass at CBM decreases monotonically. Furthermore, the color indicator also shows that all the Mg_3_Sb_2-y_Bi_y_ family members have a similar CBM between M^∗^ and L^∗^, mainly raised from the Mg3s orbital. This suggests that the alloying disordering at the Sb site might have less impact on the transport of the electron since its transport channel in the real space is around the Mg site.

**Figure 3:**
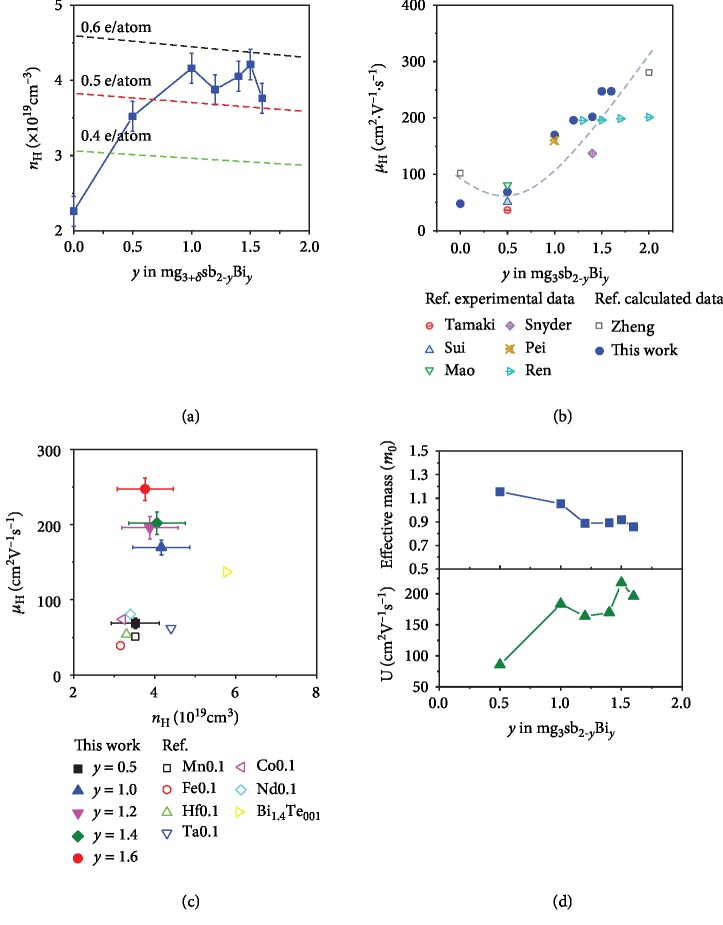
(a) Hall carrier measurement of Mg_3+*δ*_Sb_2-*y*_Bi*_y_* solid solutions (*y* = 0.5, 1, 1.2, 1.4, and 1.6). (b) Bi content-dependent Hall mobility, theoretical and experimental data from literature as references [5, 7, 8, 18, 28, 35, 39]. (c) Comparison of Hall carrier concentration and mobility in this work and reference reported Mg_3+*δ*_Sb_2-*y*_Bi*_y_* system [8, 38–40]. (d) Weighted mobility and electron effective mass of Mg_3+*δ*_Sb_2-*y*_Bi_*y*-0.01_Te_0.01_:Mn_0.01_ solid solutions (*y* = 0.5‐2.0).

Figure 3 shows the Hall measurement of the as-fabricated Mg_3+*δ*_Sb_2-*y*_Bi*_y_*, together with reported theoretical and experimental data from literature. The carrier concentrations of as-fabricated Mg_3+*δ*_Sb_2-*y*_Bi*_y_* samples show a weak dependence on Bi content in the range of *y* = 1.0‐1.6, with an average Hall carrier concentration of ~4 × 10^19^ cm^−3^, as shown in Figure 3(a). For the composition of Mg_3+*δ*_Sb_1.5_Bi_0.5_ with 1% Te and 1% Mn, our measured Hall carrier concentration (3.52 × 10^19^ cm^−3^) is slightly higher than the reported data [8, 27, 35]. The effective charge carrier per Te was estimated to be 0.30, 0.47, and 0.56 electron/atom as Bi content is *y* = 0, 0.5, and 1.0, respectively. Then, it reached a saturated value of about 0.55 electron/atom when Bi content gets larger than *y* = 1.0, which indicates that Te is a strong donor in Mg_3+*δ*_Sb_2-*y*_Bi*_y_* that is comparable with Te in CoSb_3-*x*_Te*_x_* (0.4 electron/atom) [36]. Figure 3(b) shows that the Hall mobility of as-fabricated Mg_3+*δ*_Sb_2-*y*_Bi*_y_* increases from 48.0 to 68.8, 169.8, 195.9, 201.8, 247.0, and 247.3 cm^2^ V^−1^ s^−1^ as the Bi content increases from *y* = 0 to 0.5, 1, 1.2, 1.4, 1.5, and 1.6, respectively. Based on our measured data together with theoretical and experimental ones from literature, a weak alloying effect was found [4, 5, 7, 8, 18, 35, 37–40], suggesting that the disordering Sb/Bi has a weak coupling effect on the charge transport channel. The Pisarenko curve was also used to analyze the alloying effect (Fig. [Supplementary-material supplementary-material-1], SI). The charge carrier effective masses were derived from an equivalent single band mode and changed from 1.15 to 1.05, 0.89, 0.89, 0.91, and 0.86 *m_0_* as the Bi content increased from *y* = 0.5 to 1, 1.2, 1.4, 1.5, and 1.6. It is noted that the theoretical carrier effective mass, derived from *m*^∗^ = *N*_*v*_^2/3^*m*_b_^∗^, is less than the experimental observed value, where *N*_*v*_ and *m*_b_^∗^ are the degeneration number of the CBM and band effective mass (Figures 1(d) and 2(b)). The extra disagreement between first-principles calculations and experiments could be related to the adaption of theoretical lattice constants at zero Kelvin and the screened hybrid functional HSE06. Furthermore, the conduction network forms a protected electron transport channel, away from the disordering Sb/Bi. Due to the combined effect of the electronic transport channel protection and the increased Mg_1_-Mg_2_ distance, a high Hall mobility of ~247 cm^2^ V^−1^ s^−1^ is obtained in Mg_3+*δ*_Sb_0.5_Bi_1.5_ and Mg_3+*δ*_Sb_0.4_Bi_1.6_ with 1% Te and 1% Mn samples, much higher than that of the conventional composition Mg_3+*δ*_Sb_1.5_Bi_0.5_, as shown in Figure 3(c) [36, 38–40]. Figure 3(d) shows the weighted mobility *U* = (*m*^∗^)^3/2^*μ* calculated by the carrier effective mass using the equivalent single band model and carrier mobility by Hall measurement. The weighted mobility increases from 85.5 to 183.7, 163.8, 169.7, 217.6, and 195.9 cm^2^ V^−1^ s^−1^ as the Bi content increases from *y* = 0.5 to *y* = 1.0, 1.2, 1.4, 1.5, and 1.6, respectively. This trend is consistent with our theoretical interpretation of the increasing Mg_1_-Mg_2_ distance and decreasing DOS-m^∗^ at CBM.

**Figure 4:**
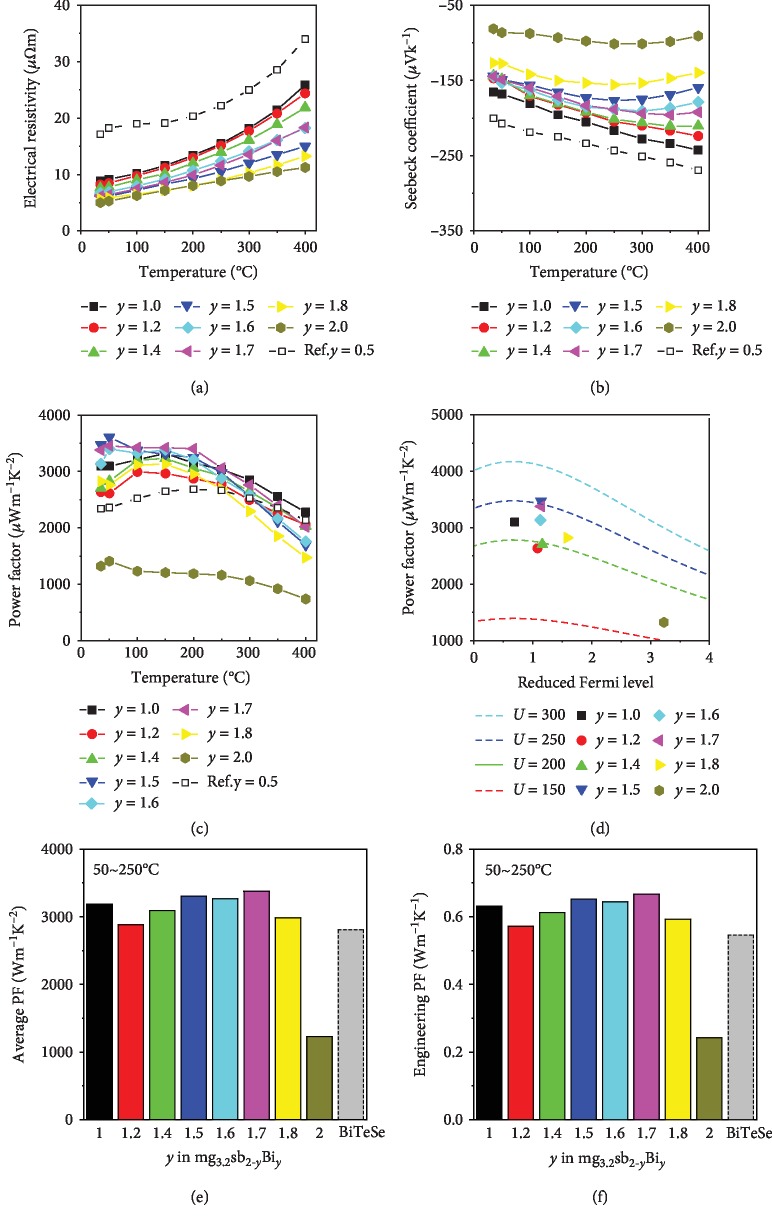
Temperature dependence of (a) electrical resistivity, (b) Seebeck coefficient, and (c) power factor of Mg_3+*δ*_Sb_2-*y*_Bi*_y_* (*y* = 1.0, 1.2, 1.4, 1.5, 1.6, 1.7, 1.8, and 2.0), previous *y* = 0.5 as reference [4]. (d) Power factor as a function of reduced Fermi level at room temperature in condition of different weighted mobility. (e) Average power factor and (f) engineering power factor in the temperature range of 50-250°C, with Bi_2_Te_2.3_Se_0.7_ as a reference [41].

Figure 4 shows the temperature-dependent electrical transport properties of as-fabricated Mg_3+*δ*_Sb_2-*y*_Bi*_y_* (*y* = 1.0‐2.0) with 1% Te and 1% Mn. The data of our previously reported Mg_3+*δ*_Sb_1.5_Bi_0.5_ is also shown for comparison [4]. Firstly, a positive correlation between temperature and electrical resistivity is found in all samples without notable abnormal negative *dρ*/*dT* near room temperature. It should be resulted from suppressed formation of Mg vacancy by using excess Mg [22] and interstitial Mn [4] and less grain boundary scattering [21] due to a large grain size of 5-10 *μ*m and high carrier concentration. The SEM images of the fracture section of as-fabricated Mg_3+*δ*_Sb_2-*y*_Bi*_y_* samples are shown in Fig. [Supplementary-material supplementary-material-1], indicating a grain size of 5-10 *μ*m. Secondly, due to the nearly unchanged carrier concentration and increasing carrier mobility with increasing Bi/Sb ratio, the room temperature electrical resistivity decreases from 8.8 to 8.2, 7.6, 6.0, 6.7, 6.2, 5.7, and 5.0 *μΩ*m as the content of Bi increases from *y* = 1.0 to *y* = 1.2, 1.4, 1.5, 1.6, 1.7, 1.8, and 2.0, respectively (Figure 4(a)). It is noted that the Mg_3+*δ*_Sb_0.5_Bi_1.5_ sample has a low electric resistivity that is only half of that of the previously reported Mg_3+*δ*_Sb_1.5_Bi_0.5_ because of its high carrier mobility. Furthermore, the room temperature Seebeck coefficient of Mg_3+*δ*_Sb_2-*y*_Bi*_y_* (*y* = 1.0‐1.7) shows a weak Bi/Sb ratio dependence (staying at almost constant around 145 *μ*V K^−1^) which is consistent with the trend of carrier concentration. As Bi content increases from *y* = 1.7 to *y* = 1.8 and 2.0, the Seebeck starts to decrease from -145 *μ*V K^−1^ to -126.7 *μ*V K^−1^ and -81.4 *μ*V K^−1^, respectively, shown in Figure 4(b). Figure 4(c) plots the temperature-dependent power factor of Mg_3+*δ*_Sb_2-*y*_Bi*_y_* (*y* = 1.0‐2.0) calculated from the measured electrical resistivity and Seebeck coefficient. All the as-fabricated Mg_3+*δ*_Sb_2-*y*_Bi*_y_* (*y* = 1.0‐2.0) samples, except for Mg_3+*δ*_Bi_2_, have a large power factor over 2500 *μ*W m^−1^ K^−2^ near room temperature. The largest room temperature power factor of 3470 *μ*W m^−1^ K^−2^ is obtained in Mg_3+*δ*_Sb_0.5_Bi_1.5_, which is 50% larger than that of our previously reported Mg_3+*δ*_Sb_1.5_Bi_0.5_ and also 17% larger than a recently reported Bi-rich Mg_3+*δ*_Sb_0.6_Bi_1.4_ (~2960 *μ*W m^−1^ K^−2^) and 26% larger than Mg_3.05_Sb_2-*x*-*y*_Bi_*y*-*x*_Te*_x_* (~2750 *μ*W m^−1^ K^−2^) [7, 8]. More comparisons were included in Fig. [Supplementary-material supplementary-material-1] (SI). Figure 4(d) plots room temperature power factor as a function of reduced Fermi level under the acoustic phonon dominant scattering (calculation details are given in SI A), suggesting that the sample Mg_3+*δ*_Sb_1.0_Bi_1.0_ was very close to the optimized carrier concentration while Mg_3+*δ*_Sb_2-*y*_Bi*_y_* (*y* = 1.2‐1.8) would be overdoped. The optimized reduced Fermi energy (*E*_F_/k_B_T) is estimated to be around 0.67, equal to *E*_F_ = 0.017 eV at room temperature, which corresponds to a Seebeck coefficient of -167 *μ*V K^−1^.

Figure 4(e) compares the average power factor of Mg_3+*δ*_Sb_2-*y*_Bi*_y_* (*y* = 1.0‐2.0) in the temperature range of 50-250°C. All the samples (except *y* = 2.0) have a value over ~3000 *μ*W m^−1^ K^−2^, which is 166% higher than Tamaki's Mg_3+*δ*_Sb_1.5_Bi_0.5_ (1130 *μ*W m^−1^ K^−2^) and also about 16% higher than that of previously reported Mg_3+*δ*_Sb_1.5_Bi_0.5_ (2590 *μ*W m^−1^ K^−2^) [4, 18]. This has already surpassed many polycrystalline n-type Bi_2_(Te_,_Se)_3_, which can be comparable with textured Bi_2_(Te_,_Se)_3_ [41, 42] and commercially available Bi_2_(Te_,_Se)_3_ ingot in the temperature range of 50-250°C. A more accurate relationship between output power density and the power factor is given in equation (1); the engineering power factor (Figure 4(f)) is calculated as equation (2) [43].

(1) $$\begin{array}{c} P_{\text{output}}={\left(\text{PF}\right)}_{\text{eng}}\frac{\Delta T}{L}\frac{\sqrt{1+{\text{ZT}}_{\text{avg}}}}{{\left(1+\sqrt{1+{\text{ZT}}_{\text{avg}}}\right)}^{2}}, \end{array}$$

(2) $$\begin{array}{c} {\left(\text{PF}\right)}_{\text{eng}}=\frac{{\left(\int_{T_{c}}^{T_{h}}S\left(T\right)dT\right)}^{2}}{\int_{T_{c}}^{T_{h}}\rho \left(T\right)dT}, \end{array}$$

where Δ*T* is the temperature difference, *L* is the TE-leg length, and *S*(*T*) and *ρ*(*T*) are temperature-dependent Seebeck coefficient and electrical resistivity. In the temperature range of 50-250°C, all the samples (except *y* = 2.0) have an engineering power factor of about 0.6 W m^−1^ K^−1^, which is 100% larger than Tamaki's Mg_3+*δ*_Sb_1.5_Bi_0.5_ (0.3 W m^−1^ K^−1^) and also 20% larger than that of previously reported Mg_3+*δ*_Sb_1.5_Bi_0.5_ (0.5 W m^−1^ K^−1^) [4, 18].

**Figure 5:**
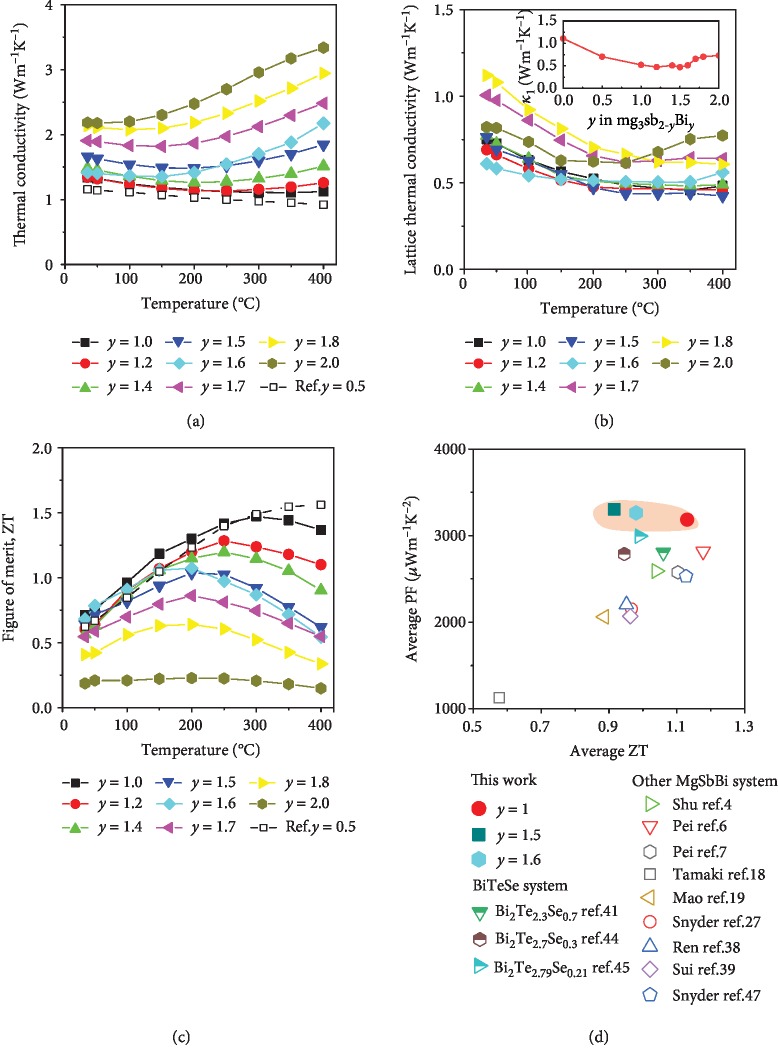
Temperature dependence of (a) thermal conductivity, (b) lattice thermal conductivity (the inset is lattice thermal conductivity as a function of Bi content at 200°C), and (c) figure of merit ZT of Mg_3+*δ*_Sb_2-*y*_Bi*_y_* (*y* = 1.0, 1.2, 1.4, 1.5, 1.6, 1.7, 1.8, and 2.0), previously reported *y* = 0.5 as reference [4, 18]. (d) Synergistic improvement of average ZT and average power factor of Mg_3+*δ*_Sb_2-*y*_Bi_*y*-0.01_Te_0.01_:Mn_0.01_ solid solutions in the temperature range of 50-250°C, compared with literature reported Mg_3+*δ*_Sb_2-*y*_Bi*_y_* and Bi_2_ (Te, Se)_3_ as reference.

Figures 5(a) and 5(b) show the temperature-dependent thermal properties of Mg_3+*δ*_Sb_2-*y*_Bi*_y_* (*y* = 1.0‐2.0). At room temperature, the thermal conductivity increases from 1.31 to 1.34, 1.47, 1.64, 1.41, 1.90, 2.13, and 2.18 W m^−1^ K^−2^, as the Bi content increases from *y* = 1.0 to 1.2, 1.4, 1.5, 1.6, 1.7, 1.8, and 2.0, respectively. A notable bipolar effect is observed in all the samples which are characterized by an increasing thermal conductivity with temperature at the high temperature end. Furthermore, the samples with more Bi (i.e., larger *y* value in the formula of Mg_3+*δ*_Sb_2-*y*_Bi*_y_*) have a lower starting temperature, which is consistent with narrowing band gap predicated by our theoretical calculations. The lattice thermal conductivity is estimated by subtracting the contribution of electronic part (*κ*_ele_) and bipolar part (*κ*_bip_) from the total thermal conductivity (*κ*_tot_), i.e., *κ*_lat_ = *κ*_tot_ − *κ*_ele_ − *κ*_bip_ (Figure 5(b)). The details of the calculation relative to the electronic thermal conductivity (*κ*_ele_) and bipolar thermal conductivity (*κ*_bip_) are given in SI B. At room temperature, *κ*_lat_ changes from 0.75 to 0.69, 0.77, 0.76, 0.61, 1.00, 1.12, and 0.82 W m^−1^ K^−2^ as the Bi content increases from *y* = 1.0 to 1.2, 1.4, 1.5, 1.6, 1.7, 1.8, and 2.0, respectively. For comparison, *κ*_lat_ of Mg_3_Sb_2_ and Mg_3_Sb_1.5_Bi_0.5_ from our previous work are estimated to be 1.46 W m^−1^ K^−2^ and 0.73 W m^−1^ K^−2^ [4]. *κ*_lat_ of Mg_3+*δ*_Sb_1.0_Bi_1.0_ is 48% lower than that of Mg_3_Sb_2_ and 12% lower than that of Mg_3_Bi_2_. This decrease in lattice thermal conductivity is the result of alloying scattering on the transport of the phonon.

Figure 5(c) compares ZT as a function of temperature of Mg_3+*δ*_Sb_2-*y*_Bi*_y_* (*y* = 1.0‐2.0). Mg_3+*δ*_Sb_1.0_Bi_1.0_ shows a peak ZT of 1.5 at 300°C, while a room temperature ZT of 0.75_,_ which is 275% higher than that of Tamaki's Mg_3+*δ*_Sb_1.5_Bi_0.5_ (ZT_@RT_ = 0.2) and 20% higher than that of our previously reported Mg_3+*δ*_Sb_1.5_Bi_0.5_ (ZT_@RT_ = 0.62), as shown in Figure 5(c) [4, 18]. This is also comparable with recently reported Mg_3.2_Bi_1.998-*x*_Sb*_x_*Te_0.002_ at room temperature [5] and higher than Mg_3+*δ*_Sb_0.6_Bi_1.4_, which behaves ZT_@RT_~0.68 and peak ZT of ~1.1 [8]. It is comparable with those of commercially available n-type Bi_2_Te_3_ ingot (ZT_@RT_ = 0.71) [4]. Moreover, in the temperature range of 50-250°C, Mg_3+*δ*_Sb_1.0_Bi_1.0_ has an engineering ZT of 0.56, which is 16.7% higher than our previously reported Mg_3+*δ*_Sb_1.5_Bi_0.5_ ((ZT)_eng_ = 0.48) and Bi_2_Te_3-*x*_Se*_x_* ((ZT)_eng_ = 0.47) (data from literatures are plotted on Fig. [Supplementary-material supplementary-material-1], SI) [4, 44]. Furthermore, the thermoelectric properties of three batches of Mg_3+*δ*_Sb_1.0_Bi_1.0_ and circling electrical property test for one of the samples were given in Fig. [Supplementary-material supplementary-material-1] (SI), which shows that the results are repeatable. Finally, a dual parameter criteria of “ZT versus PF” [43] are used to select the better thermoelectric materials with a consideration for requirements of efficiency and power density at the same time [45]. Figure 5(d) clearly suggests that Mg_3+*δ*_Sb_1.0_Bi_1.0_ in this work surpasses any other Mg_3+*δ*_Sb_2-*y*_Bi*_y_* material in this work and the Bi_2_Te_3-*x*_Se*_x_* family [4, 6, 7, 18, 19, 27, 38, 39, 41, 44, 46, 47], for its high average ZT of 1.13 and an average power factor of 3184 *μ*W m^−1^ K^−2^. (Reference data from literature is plotted on Fig. [Supplementary-material supplementary-material-1], SI.).

## 3. Conclusion

We have successfully enhanced the room temperature thermoelectric performance of Mg_3+*δ*_Sb_2-*y*_Bi*_y_* by the strategy of electronic transport channel protection and tuning in real space. It was found that the increased carrier mobility was closely related to the weakening covalence-like bonding between Mg_1_ 3s and Mg_2_ 3s orbitals and hence the lightening DOS effective mass. Experimentally, our Mg_3+*δ*_Sb_0.5_Bi_1.5_ samples reached a high carrier mobility of 247 cm^2^ V^−1^ s^−1^ and high power of 3470 *μ*W m^−1^ K^−2^ at room temperature. We also suggest that, in a dual parameter criteria of “ZT versus PF,” Mg_3+*δ*_Sb_1.0_Bi_1.0_ would be a promising room temperature thermoelectric material, to replace the classic n-type Bi_2_Te_2.7_Se_0.3_, for its high values of ZT_@RT_ = 0.75, PF_@RT_ = 3100 *μ*W m^−1^ K^−2^ at room temperature, (ZT)_avg_ = 1.13, and (PF)_avg_ = 3184 *μ*W m^−1^ K^−2^ in the temperature range of 50-250°C, which gives an efficiency of 8.5% under ideal adiabatic condition. Furthermore, the electronic transport channel protection and tuning in real space could be a new electronic engineering strategy to increase the carrier mobility.

## 4. Experimental Procedures

### 4.1. Sample Synthesis

The samples with nominal compositions of Mg_3+*δ*_Sb_2-*y*_Bi_*y*-0.01_Te_0.01_:Mn_0.01_ were synthesized by mechanical alloying and spark plasma sintering (SPS). High-purity magnesium turnings (Mg, >99.9%; Acros Organics), antimony shots (Sb, 99.999%; 5N Plus), bismuth shots (Bi, 99.999%; 5N Plus), tellurium shots (Te, 99.999%; 5N Plus), and manganese powders (Mn, 99.95%; Alfa Aesar) were weighed according to the composition of Mg_3+*δ*_Sb_2-*y*_Bi_*y*-0.01_Te_0.01_:Mn_0.01_ (*δ* = 0.2, *y* = 1.0, 1.2, 1.4, 1.5, 1.6, 1.7, 1.8, and 2.0), simplified as Mg_3+*δ*_Sb_2-*y*_Bi*_y_* in the text, and were then loaded into a stainless steel ball milling jar together with stainless steel balls in a glove box in an argon atmosphere with the oxygen level < 1 ppm. After ball milling for 8 hours in SPEX 8000D, or 8000 M, the ball-milled powders were loaded into a graphite die with an inner diameter of 15 mm in a glove box. Graphite die with loading powder was immediately sintered at 675°C under a pressure of 50 MPa for 5 min in SPS division (SPS-211Lx, Fuji Electronic Industrial Co. LTD). The SPS bulks are ~15 mm in diameter and ~8 mm in thickness. The Seebeck coefficient, electrical resistivity, and thermal diffusivity were measured in the directions perpendicular to pressure.

### 4.2. Thermoelectric Characterization

Electrical properties, including Seebeck coefficient, electrical resistivity, and power factor, were measured by ZEM-3, ULVAC Riko, under a 0.01 MPa pressure helium atmosphere from RT to 400°C. Measured samples were cut into about 2.5 mm × 3 mm × 13 mm pieces. Thermal conductivity was calculated by equation *κ* = *DC*_*p*_*ρ*, where *D* is thermal diffusivity measured by laser flash method (LFA 467; Netzsch) using about 6 mm × 6 mm × 1 mm pieces, *d* is density measured by the Archimedean method, and specific heat (*C*_*p*_) is tested by differential scanning calorimetry (Discovery DSC, Waters LLC), shown in Fig. [Supplementary-material supplementary-material-1] (SI).

#### 4.2.1. Hall Effect Measurement

Hall coefficient was measured by Physical Property Measurement System (PPMS-14L, Quantum Design) with four-point method in magnetic field from -5 T to 5 T. Tested samples were cut into about 6 mm × 6 mm × 1 mm pieces and then soldered to *Φ*0.1 mm enameled wire with In as the solder. Hall carrier concentration was calculated by *n*_*H*_ = 1/|*R*_*H*_|*e*, and Hall mobility was calculated by *μ*_*H*_ = |*R*_*H*_|/*ρ*, where *e* is elementary charge and *ρ* is measured electrical resistivity.

### 4.3. X-Ray Diffraction

SPS bulks were grinded into powder in a glove box, and then, the phase composition was characterized by X-ray diffraction (Rigaku SmartLab) with Cu K*_α_* radiation (*λ* = 1.54 Å, operating at 40 kV/15 mA with *K_β_* foil filter). XRD patterns were further refined by the Rietveld method to calculate lattice parameter and Mg_1_-Mg_2_ distance.

### 4.4. Calculation Methods

The first-principles calculations are based on density functional theory (DFT) [48, 49] and the screened hybrid functional HSE06 [50, 51] as implemented in the Vienna Ab Initio Simulation Package (VASP) code [52]. Projected augmented wave (PAW) potentials [53] with plane-wave basis set and an energy cutoff of 550 eV are used. The valence electronic configurations for Mg, Sb, and Bi are 3s^2^, 5s^2^5p^3^, and 6s^2^6p^3^ in the pseudopotentials, respectively. For integrations over the Brillouin zone, we use 5 × 5 × 3 Monkhorst-Pack k-point mesh [54] for 40-atom cells (2 × 2 × 2). The atomic positions are fully relaxed until the forces on each atom are less than 0.005 eV/Å and total energy differences between two consecutive steps are less than 10^−6^ eV. The lowest-energy structural configurations for alloy systems are constructed by Supercell program [55] and direct energy comparison.

## Figures and Tables

**Table 1:** First-principles calculation for Mg_3_Sb_2-*y*_Bi*_y_*.

*y*	Band gap at *Γ*-CBM (eV)	Band gap at *Γ*-K (eV)	Effective mass at CBM (*m*_0_)	Lattice constant *a* (Å)	Lattice constant *c* (Å)	Mg_1_-Mg_2_ distance (Å)
0.00	0.50	0.72	0.139	4.596	7.276	3.770
0.50	0.39	0.68	0.123	4.625	7.324	3.801
0.75	0.33	0.66	0.114	4.639	7.344	3.817
1.00	0.28	0.64	0.107	4.644	7.363	3.832
1.25	0.22	0.62	0.102	4.664	7.387	3.847
1.50	0.17	0.61	0.097	4.681	7.409	3.862
2.00	0.14	0.58	0.083	4.718	7.453	3.891

## Supplementary Materials

Supplementary Materials Fig. S1: XRD patterns of the Mg_3+*δ*_Sb_2-*y*_Bi*_y_* (*y* = 1.0, 1.2, 1.4, 1.5, 1.6, 1.7, 1.8, and 2.0) solid solution powder. Fig. S2: lattice parameter *a* and *c* of Mg_3+*δ*_Sb_2-*y*_Bi*_y_* as function of Bi content, and dash lines show Vegard's law between Mg_3_Sb_2_ and Mg_3_Bi_2_. Fig. S3: Pisarenko's plot for Mg_3+*δ*_Sb_2-*y*_Bi*_y_*. It shows a decrease effective mass with higher Bi content. Fig. S4: SEM images of the fractured surface of Mg_3+*δ*_Sb_2-*y*_Bi*_y_*: (a) *y* = 1.0, (b) *y* = 1.2, (c) *y* = 1.4, (d) *y* = 1.5, and (e) *y* = 1.6. Fig. S5: comparison of power factor of the Mg_3_ (Sb, Bi)_2_ system [1–18]. Fig. S6: reproducibility and circling test of as-fabricated Mg_3+*δ*_Sb_1.0_Bi_0.99_Te_0.01_:Mn_0.01_. Fig. S7: temperature dependence of (a) power factor and (b) ZT of as-fabricated Mg_3+*δ*_Sb_2-*y*_Bi*_y_* and Bi_2_(Te,Se)_3_ from references [8, 9, 12, 15, 17–23]. Fig. S8: engineering ZT of Mg_3+*δ*_Sb_2-*y*_Bi_*y*-0.01_Te_0.01_:Mn_0.01_ in the temperature range of 50-250°C. Fig. S9: specific heat of the Mg_3+*δ*_Sb_2-*y*_Bi_*y*-0.01_Te_0.01_:Mn_0.01_ solid solution powder.
