# Supplementary material for: The Electronic Transport Channel Protection and Tuning in Real Space to Boost the Thermoelectric Performance of Mg3+δSb2-yBiy near Room Temperature
Source: Research (Wash D C). 2020 Feb 28;2020:1672051. doi: 10.34133/2020/1672051 (PMC7064820; doi:10.34133/2020/1672051)
Supplement: Supplementary Materials — Fig. S1: XRD patterns of the Mg3+δSb2-yBiy (y = 1.0, 1.2, 1.4, 1.5, 1.6, 1.7, 1.8, and 2.0) solid solution powder. Fig. S2: lattice parameter a and c of Mg3+δSb2-yBiy as function of Bi content, and dash lines show Vegard's law between Mg3Sb2 and Mg3Bi2. Fig. S3: Pisarenko's plot for Mg3+δSb2-yBiy. It shows a decrease effective mass with higher Bi content. Fig. S4: SEM images of the fractured surface of Mg3+δSb2-yBiy: (a) y = 1.0, (b) y = 1.2, (c) y = 1.4, (d) y = 1.5, and (e) y = 1.6. Fig. S5: comparison of power factor of the Mg3 (Sb, Bi)2 system [1–18]. Fig. S6: reproducibility and circling test of as-fabricated Mg3+δSb1.0Bi0.99Te0.01:Mn0.01. Fig. S7: temperature dependence of (a) power factor and (b) ZT of as-fabricated Mg3+δSb2-yBiy and Bi2(Te,Se)3 from references [8, 9, 12, 15, 17–23]. Fig. S8: engineering ZT of Mg3+δSb2-yBiy-0.01Te0.01:Mn0.01 in the temperature range of 50-250°C. Fig. S9: specific heat of the Mg3+δSb2-yBiy-0.01Te0.01:Mn0.01 solid solution powder. [file 1672051.f1.docx]

Supplementary Materials

for

The electronic transport channel protection and tuning in real space to boost the thermoelectric performance of Mg_3+δ_Sb_2-y_Bi_y_ near room temperature

**Zhijia Han,^a,c†^ Zhigang Gui,^b, d†^ Y.B. Zhu,^a^ Peng Qin,^a^ Bo-Ping Zhang,^c^ Wenqing Zhang,^b^ Li Huang^b*^ and Weishu Liu ^a, e*^**

a. Department of Materials Science and Engineering, Southern University of Science and Technology, Shenzhen 518055, China.

b. Department of Physics, Southern University of Science and Technology, Shenzhen 518055, China

c. School of Materials Science and Engineering, University of Science and Technology Beijing, Beijing 10083, China

d. Academy for Advanced Interdisciplinary Studies, Southern University of Science and Technology, Shenzhen 518055, China

e. Shenzhen Engineering Research Center for Novel Electronic Information Materials and Devices, Southern University of Science and Technology, Shenzhen 518055, China.

† Equivalent contribution.

* Email: liuws@ sustech.edu.cn，huangl@ sustech.edu.cn

**Fig. S1.** XRD patterns of the Mg_3+δ_Sb_2-y_Bi_y_ (y=1.0, 1.2, 1.4, 1.5, 1.6, 1.7, 1.8, 2.0) solid solutions powder


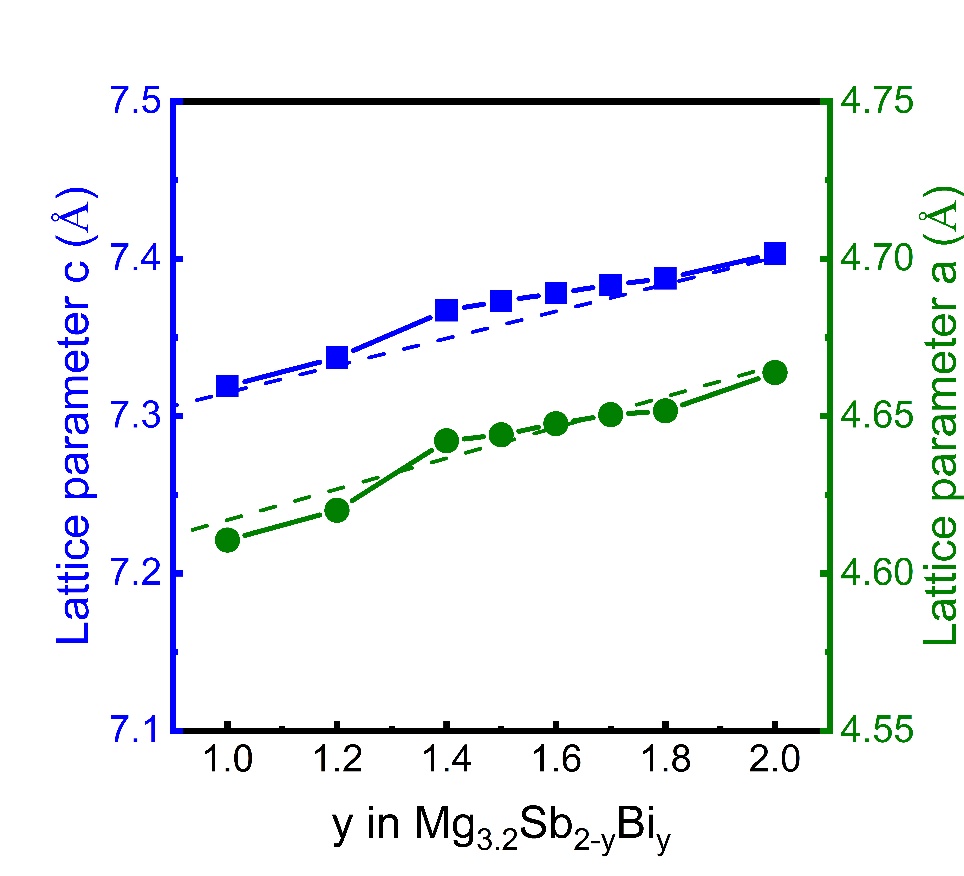


**Fig. S2.** Lattice parameter a and c of Mg_3+δ_Sb_2-y_Bi_y_ as function of Bi content, and dash lines show Vegard’s law between Mg_3_Sb_2_ and Mg_3_Bi_2_


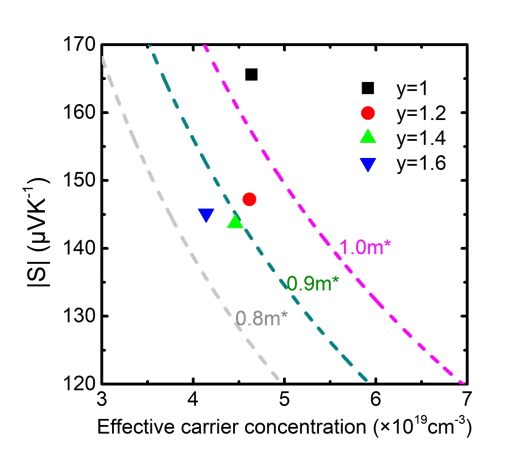


**Fig. S3.** Pisarenko’s plot for the Mg_3+δ_Sb_2-y_Bi_y_. It shows a decrease effective mass with higher Bi content.


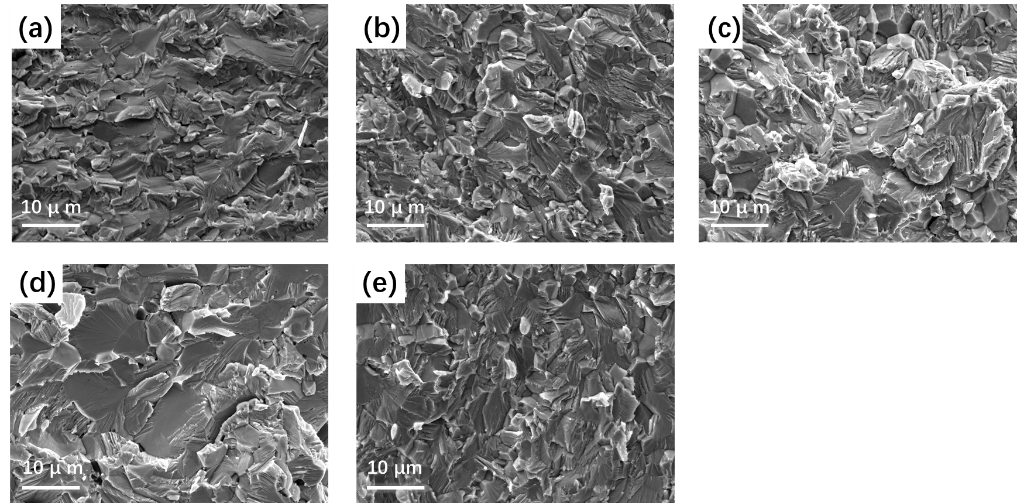


**Fig. S3** SEM images of the fractured surface of the Mg_3+δ_Sb_2-y_Bi_y_:

(a) y = 1.0, (b) y = 1.2, (c) y = 1.4, (d) y = 1.5, (e) y = 1.6

**Fig. S5.** Comparison of power factor of the Mg_3_(Sb,Bi)_2_ system[1-18]


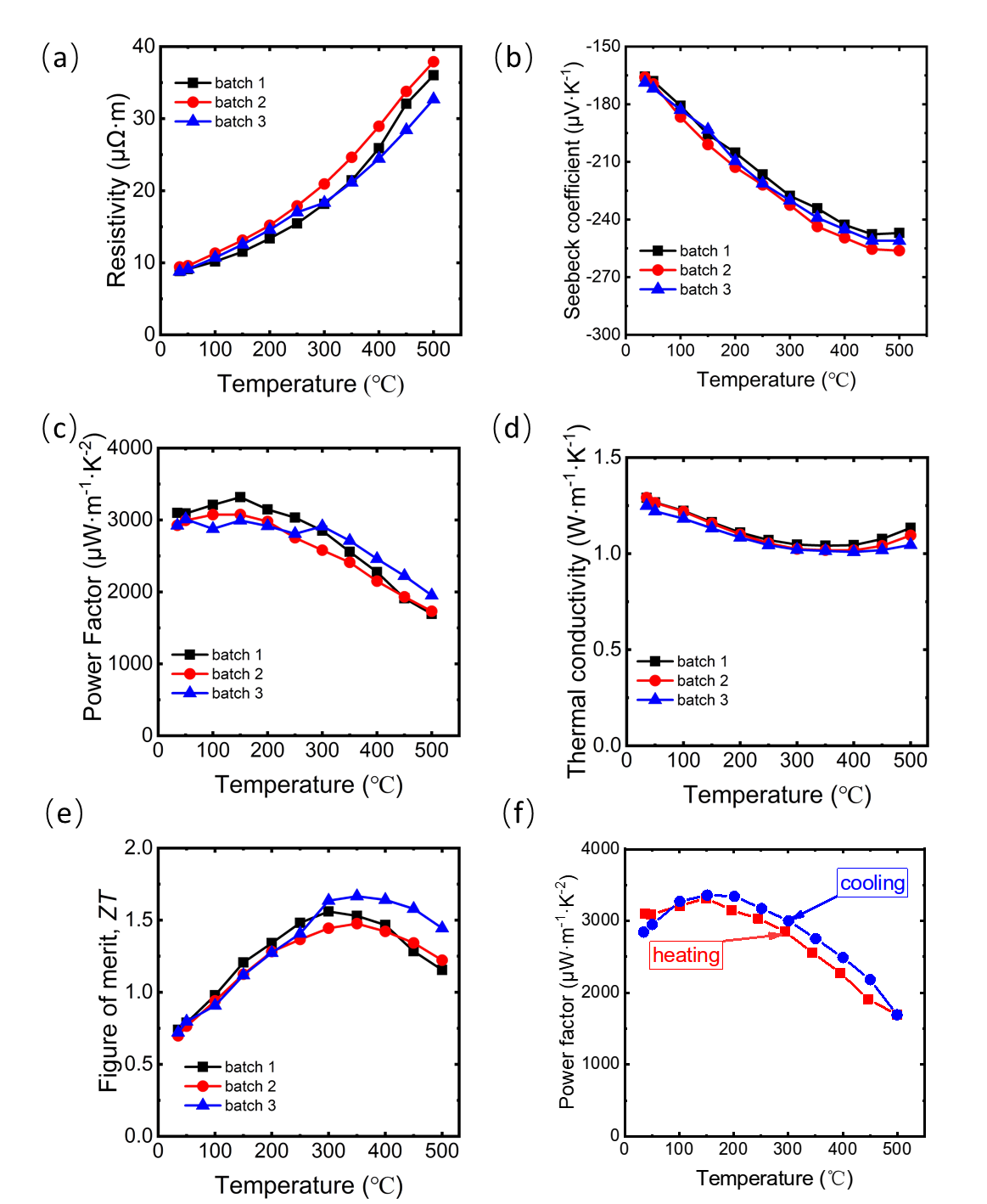


**Fig. S6.** Reproducibility and circling test of as-fabricated Mg_3+δ_Sb_1.0_Bi_0.99_Te_0.01_:Mn_0.01_.


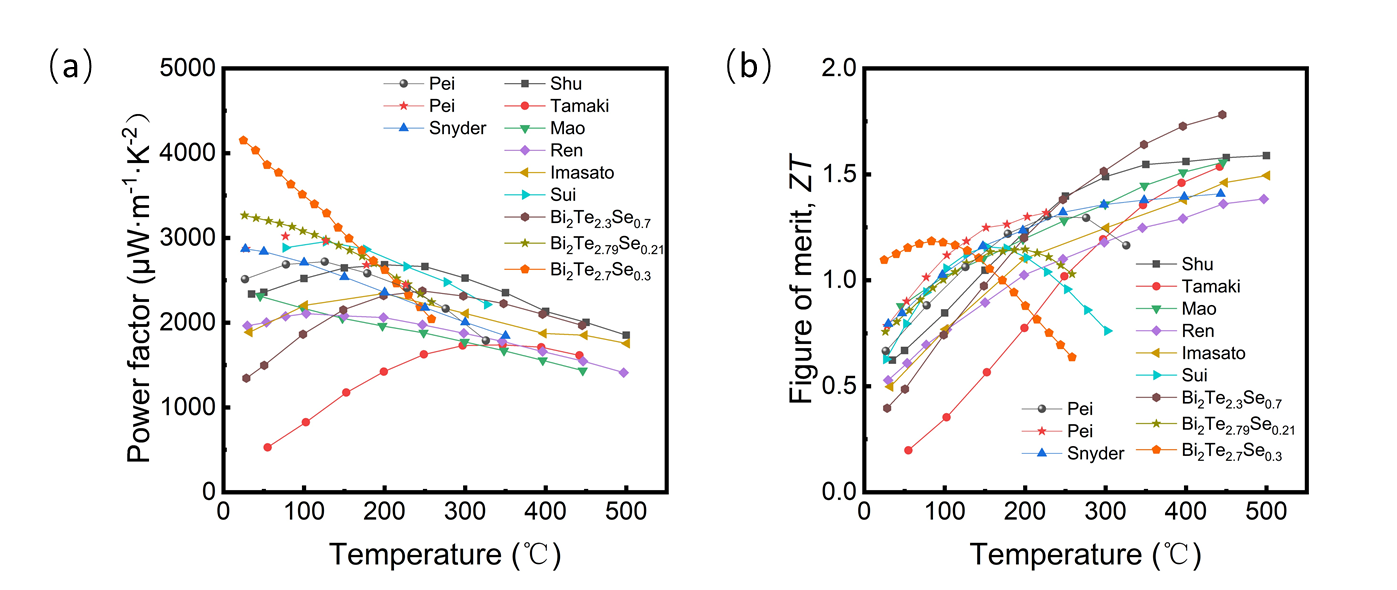


**Fig. S7.** Temperature dependence of (a) power factor and (b)*ZT* of as-fabricated Mg_3+δ_Sb_2-y_Bi_y_ and Bi_2_(Te,Se)_3_ from references[8, 9, 12 , 15, 17-23]


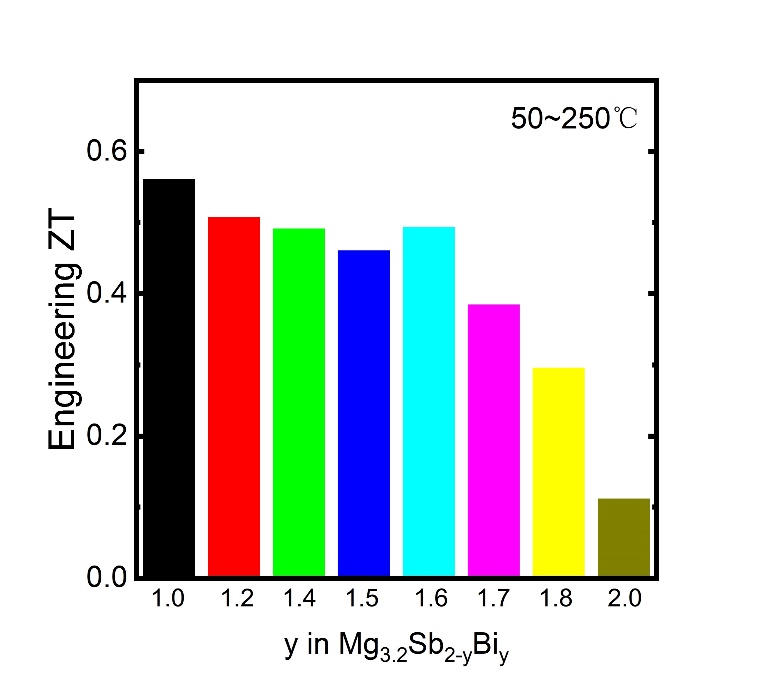


**Fig. S8.** Engineering ZT of the Mg_3+δ_Sb_2-y_Bi_y-0.01_Te_0.01_:Mn_0.01_ in temperature range of 50-250 ^o^C


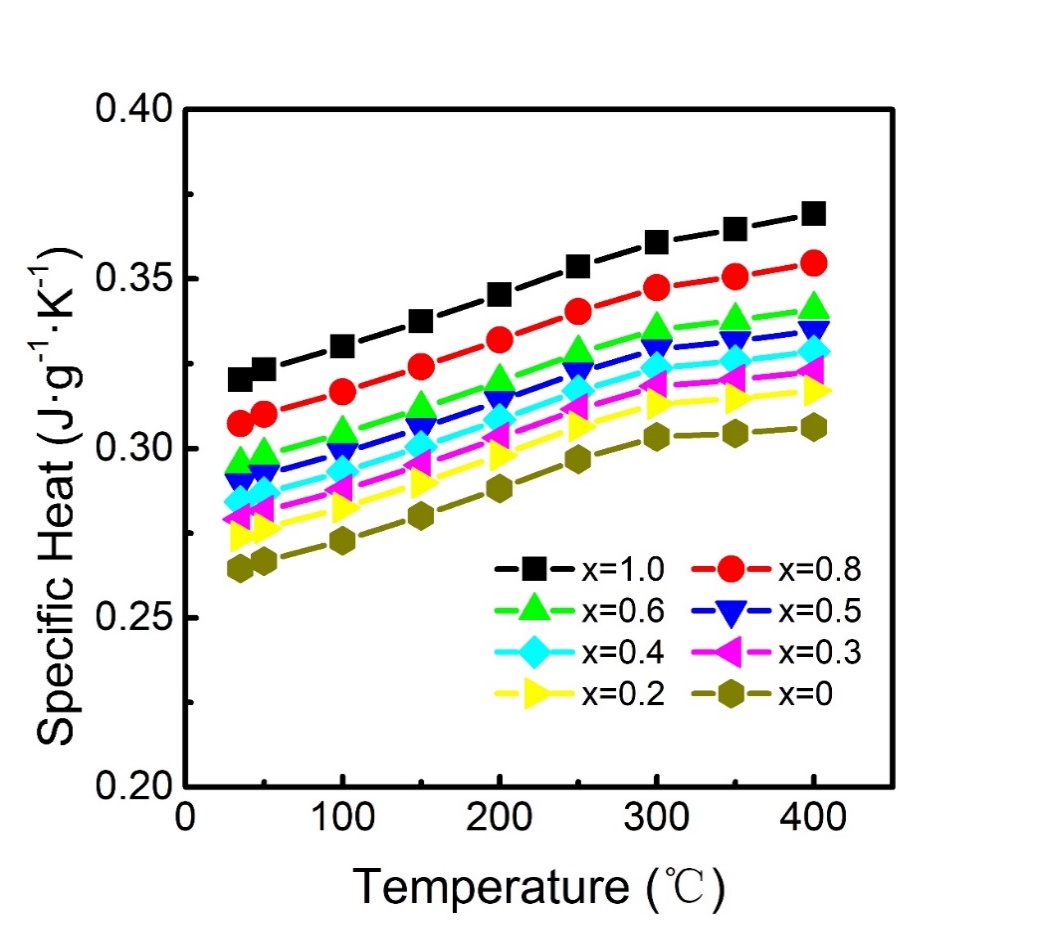


**Fig. S9.** Specific heat of the Mg_3+δ_Sb_2-y_Bi_y-0.01_Te_0.01_:Mn_0.01_ solid solutions powder.

**A: Expression of power factor in condition of acoustic scattering**

| $S=\text{-}\frac{k_{B}}{e}\left(\frac{2F_{1}\left(\xi\right)}{F_{0}\left(\xi\right)}-\xi\right)$ | (S13) |
| --- | --- |
| $n=\frac{2\left(2\pi m_{d}^{*}k_{B}T\right)^{3/2}}{h^{3}}F_{0}\left(\xi\right)$ | (S14) |
| $\mu=\frac{eh^{4}\rho_{d}}{6\pi\Delta^{2}\left(2m_{b}^{*}\right)^{5/2}E^{1/2}}$ | (S15) |
| where *ρ_d_* is density, *Δ* is deformation potential constant, and *E* is longitudinal elastic constant. Considering the relationship of $m_{d}^{*}=\left(N_{V}\right)^{2/3}m_{b}^{*}$ and $U=\left(\frac{m*}{m_{0}}\right)^{3/2}\mu$, | |
| $\begin{matrix} PF=S^{2}/\rho=S^{2}ne\mu\\ =\left(\frac{k_{B}}{e}\right)^{2}\left(\frac{2F_{1}\left(\xi\right)}{F_{0}\left(\xi\right)}-\xi\right)^{2}\frac{2\left(2\pi m_{d}^{*}k_{B}T\right)^{3/2}}{h^{3}}F_{0}\left(\xi\right)\frac{eh^{4}\rho_{d}}{6\pi\Delta^{2}\left(2m_{b}^{*}\right)^{5/2}E^{1/2}} \\ =\left(\frac{k_{B}}{e}\right)^{2}\left(\frac{2F_{1}\left(\xi\right)}{F_{0}\left(\xi\right)}-\xi\right)^{2}\frac{2\left(2\pi k_{B}T\right)^{3/2}}{h^{3}}F_{0}\left(\xi\right)\frac{eh^{4}\rho_{d}}{6\pi\Delta^{2}\left(2\right)^{5/2}E^{1/2}}\frac{N_{V}}{m_{b}^{*}} \\ or=\left(\frac{k_{B}}{e}\right)^{2}\left(\frac{2F_{1}\left(\xi\right)}{F_{0}\left(\xi\right)}-\xi\right)^{2}\frac{2\left(2\pi m_{d}^{*}k_{B}T\right)^{3/2}}{h^{3}}F_{0}\left(\xi\right)\mu\\ =\left(\frac{k_{B}}{e}\right)^{2}\left(\frac{2F_{1}\left(\xi\right)}{F_{0}\left(\xi\right)}-\xi\right)^{2}\frac{2\left(2\pi m_{0}k_{B}T\right)^{3/2}}{h^{3}}F_{0}\left(\xi\right)U \end{matrix}$ | (S16) |

Here, draw a conclusion that power factor is proportional to the ratio *Nv/m** and weighted mobility *U*.

**B: Calculate lattice thermal conductivity**

Lattice thermal conductivity was calculated by $\kappa_{lat}=\kappa_{tot}-\kappa_{ele}-\kappa_{bip}$, where $\kappa_{lat}$ is lattice thermal conductivity, $\kappa_{tot}$ is measured total thermal conductivity, $\kappa_{ele}$ is electron thermal conductivity, and $\kappa_{bip}$is bipolar thermal conductivity, respectively.

**B1:** Calculate bipolar thermal conductivity.

The room temperature reduced Fermi energy is derived from both the carrier concentration and Seebeck coefficient on the basis of single band approximation, as Equation 1 and Equation 2.

| $n=4\pi\left(\frac{2m^{*}k_{B}T}{h^{2}}\right)^{3/2}F_{1/2}\left(\xi\right)$ | (S1) |
| --- | --- |
| $S=\pm\frac{k_{B}}{e}\left(\frac{\left(r+5/2\right)F_{r+3/2}\left(\xi\right)}{\left(r+3/2\right)F_{r+1/2}\left(\xi\right)}-\xi\right)$ | (S2) |

where *m** is carrier effective mass, *k_B_* is Boltzmann constant, *e* is elementary charge, *ξ* is reduced Fermi level, *h* is plank constant, *r* is scattering parameter, and *F_n_(ξ)* is Fermi integrate defined as $F_{n}(\xi)=\int_{0}^{\infty} \frac{x^{n}}{1+e^{(x-\xi)}}\text{d}x$

Assuming the intrinsic carrier *n_0_* is negligible and external doping is fully excitation, external dopant *N_D_* is equal to be carrier concentration at room temperature. And then, from Equation 3-5, electron concentration, hole concentration and reduced Fermi energy at whole temperature range can be solved.

| $n=p_{0}+N_{D}$ | (S3) |
| --- | --- |
| $n=\frac{2}{\sqrt{\pi}}N_{C}F_{1/2}\left(\xi\right)=4\pi\left(\frac{2m_{e}^{*}k_{B}T}{h^{2}}\right)^{3\text{/2}}F_{1/2}\left(\xi\right)$ | (S4) |
| $p=\frac{2}{\sqrt{\pi}}N_{V}F_{1/2}\left(\xi\right)=4\pi\left(\frac{2m_{h}^{*}k_{B}T}{h^{2}}\right)^{3\text{/2}}F_{1/2}\left(-\xi-\xi_{G}\right)$ | (S5) |

where *ξ_G_* is band gap.

By Equation 6-7 and reduced Fermi energy solved above, Seebeck coefficient caused by different carrier *S_n_* and *S_p_* can be confirmed.

| $S_{n}=\pm\frac{k_{B}}{e}\left(\frac{\left(r+5/2\right)F_{r+3/2}\left(\xi\right)}{\left(r+3/2\right)F_{r+1/2}\left(\xi\right)}-\xi\right)$ | (S6) |
| --- | --- |
| $S_{p}=\pm\frac{k_{B}}{e}\left(\frac{\left(r+5/2\right)F_{r+3/2}\left(-\xi-\xi_{G}\right)}{\left(r+3/2\right)F_{r+1/2}\left(-\xi-\xi_{G}\right)}+\xi+\xi_{G}\right)$ | (S7) |

After that, electron conductivity *σ_n_* and hole conductivity *σ_p_* can be determined by Equation 8-10

| $\sigma_{p}=Ap_{0}\mu_{aver}e$ | (S8) |
| --- | --- |
| $\sigma_{n}=n_{0}\mu_{aver}e$ | (S9) |
| $\sigma_{n}S_{n}+\sigma_{p}S_{p}=\sigma S$ | (S10) |

where *μ_aver_* is average mobility, and A is a parameter to fit calculated Seebeck coefficient and measured Seebeck coefficient. Finally, bipolar thermal conductivity is calculated by Equation 11.

| $\kappa_{bip}=\frac{\sigma_{n}\sigma_{p}}{\sigma_{n}+\sigma_{p}}\left(S_{n}-S_{p}\right)^{2}T$ | (S11) |
| --- | --- |

where *T* is absolute temperature.

Effective mass and band gap used in above calculation is listed in **Table S1**.

**Table S1.** parameters used in bipolar thermal conductivity calculation

| y in Mg_3_Sb_2-y_Bi_y_ | Electron effective mass *m_e_**(m_0_) | Hole effective mass *m_h_**(m_0_) | Band gap *ξ_G_*(eV) | d*ξ_G_*/dT  (×10^-5^eV K^-1^) |
| --- | --- | --- | --- | --- |
| 1.0 | 1.05 | 0.75 | 0.2245 | 1.16 |
| 1.2 | 0.93 | 0.75 | 0.2091 | 0.70 |
| 1.4 | 0.89 | 0.75 | 0.1928 | 0.43 |
| 1.5 | 0.87 | 0.75 | 0.1846 | 0.33 |
| 1.6 | 0.86 | 0.75 | 0.1765 | 0.26 |
| 1.7 | 0.82 | 0.75 | 0.1764 | 0.20 |
| 1.8 | 0.75 | 0.75 | 0.1439 | 0.16 |
| 2.0 | 0.20 | 0.75 | 0.0660 | 0.09 |

In addition, several theoretical works predicted that the Mg_3_Bi_2_ was zero band gap topologic semimetal. However, in this work, band gap of Mg_3_Bi_2_ was calculated by HSE is still a small positive value. And it’s worthy to point out that Ren *et al.* calculated band gap of Mg_3_Bi_1.9_Sb_0.1_ as a positive value while band gap of Mg_3_Bi_2_ is a small negative value.[24]

**B2:** Calculate electron thermal conductivity**.**

By inject calculated reduced Fermi energy into Equation 12, Lorenz constant(*L*) in whole temperature range can be calculated, and then electron thermal conductivity by $\kappa_{ele}=L\sigma T$.

| $L=\left(\frac{k_{B}}{e}\right)^{2}\left(\frac{\left(r+7\text{/2}\right)F_{r+5/2}\left(\xi\right)}{\left(r+3\text{/2}\right)F_{r+1/2}\left(\xi\right)}-\left[\frac{\left(r+5\text{/2}\right)F_{r+3/2}\left(\xi\right)}{\left(r+3\text{/2}\right)F_{r+1/2}\left(\xi\right)}\right]^{2}\right)$ | (S12) |
| --- | --- |

**References**

1. S. Kim, C. Kim, Y. K. Hong, T. Onimaru, K. Suekuni, T. Takabatake, M. H. Jung. Thermoelectric properties of Mn-doped Mg–Sb single crystals. *Journal of Materials Chemistry A*, vol. 2, no. 31, pp. 12311-12316, 2014.
2. A. Bhardwaj, A. Rajput, A. Shukla, J. Pulikkotil, A. Srivastava, A. Dhar, G. Gupta, S. Auluck, D. Misra, R. Budhani. Mg_3_Sb_2_-based Zintl compound: a non-toxic, inexpensive and abundant thermoelectric material for power generation. *RSC Advances*, vol. 3, no. 22, pp. 8504-8516, 2013.
3. V. Ponnambalam, D. T. Morelli. On the thermoelectric properties of Zintl compounds Mg_3_Bi_2− x_Pn_x_ (Pn= P and Sb). *Journal of electronic materials*, vol. 42, no. 7, pp. 1307-1312, 2013.
4. F. Ahmadpour, T. Kolodiazhnyi, Y. Mozharivskyj. Structural and physical properties of Mg_3− x_Zn_x_Sb_2_ (x= 0–1.34). *Journal of Solid State Chemistry*, vol. 180, no. 9, pp. 2420-2428, 2007.
5. A. Bhardwaj, A. Shukla, S. Dhakate, D. Misra. Graphene boosts thermoelectric performance of a Zintl phase compound. *Rsc Advances*, vol. 5, no. 15, pp. 11058-11070, 2015.
6. J. Shuai, Y. M. Wang, H. S. Kim, Z. H. Liu, J. Y. Sun, S. Chen, J. H. Sui, Z. F. Ren. Thermoelectric properties of Na-doped Zintl compound: Mg_3− x_Na_x_Sb_2_. *Acta Materialia*, vol. 93, pp. 187-193, 2015.
7. J. W. Zhang, L. R. Song, S. H. Pedersen, H. Yin, L. T. Hung, B. B. Iversen. Discovery of high-performance low-cost n-type Mg_3_Sb_2_-based thermoelectric materials with multi-valley conduction bands. *Nature communications*, vol. 8, pp. 13901, 2017.
8. H. Tamaki, H. K Sato, T. Kanno. Isotropic Conduction Network and Defect Chemistry in Mg_3+δ_Sb_2_‐Based Layered Zintl Compounds with High Thermoelectric Performance. *Advanced Materials*, vol. 28, no. 46, pp. 10182-10187, 2016.
9. J. Mao, Y. X. Wu, S. W. Song, Q. Zhu, J. Shuai, Z. H. Liu, Y. Z. Pei, Z. F. Ren. Defect engineering for realizing high thermoelectric performance in n-Type Mg_3_Sb_2_-based materials. *ACS Energy Letters*, vol. 2, no. 10, pp. 2245-2250, 2017.
10. J. Mao, J. Shuai, S. W. Song, Y. X. Wu, R. Dally, J. W. Zhou, Z. H. Liu, J. F. Sun, Q. Y. Zhang, C. dela Cruz, S. wilson, Y. Z. Pei, D. J. Singh, G. Chen, C.-C. Chu, Z. F. Ren. Manipulation of ionized impurity scattering for achieving high thermoelectric performance in n-type Mg_3_Sb_2_-based materials. *Proceedings of the National Academy of Sciences*, vol. 114, no. 40, pp. 10548-10553, 2017.
11. J. W. Zhang, L. R. Song, A. Mamakhel, M. R. V. Jørgensen, B. B. Iversen. High-performance low-cost n-type Se-doped Mg_3_Sb_2_-based Zintl compounds for thermoelectric application. *Chemistry of Materials*, vol. 29, no. 12, pp. 5371-5383, 2017.
12. S. W. Song, J. Mao, J. Shuai, H. T. Zhu, Z. S. Ren, U. Saparamadu, Z. J. Tang, B. Wang, Z. F. Ren. Study on anisotropy of n-type Mg_3_Sb_2_-based thermoelectric materials. *Applied Physics Letters*, vol. 112, no. 9, pp. 092103, 2018.
13. T. Kanno, H. Tamaki, H. K. Sato, S. D. Kang, S. Ohno, K. Imasato, J. J. Kuo, G. J. Snyder, Y. Miyazaki. Enhancement of average thermoelectric figure of merit by increasing the grain-size of Mg_3. 2_Sb_1. 5_Bi_0. 49_Te_0. 01_. *Applied Physics Letters*, vol. 112, no. 3, pp. 033903, 2018.
14. J. Shuai, J. Mao, S. W. Song, Q. Zhu, J. F. Sun, Y. M. Wang, R. He, J. W. Zhou, G. Chen, D. J. Singh, Z. F. Ren. Tuning the carrier scattering mechanism to effectively improve the thermoelectric properties. *Energy & Environmental Science*, vol. 10, no. 3, pp. 799-807, 2017.
15. K. Imasato, S. D. Kang, G. J. Snyder. Exceptional thermoelectric performance in Mg_3_Sb_0.6_Bi_1.4_ for low-grade waste heat recovery. *Energy & Environmental Science*, vol. 12, no. 3, pp. 965-971, 2019.
16. Y. T. Liu, C. G. Fu, K. Y. Xia, J. J. Yu, X.B. Zhao, H. G. Pan, C. Felser, T. J. Zhu. Lanthanide Contraction as a Design Factor for High‐Performance Half‐Heusler Thermoelectric Materials. *Advanced Materials*, vol. 30, no. 32, pp. 1800881, 2018.
17. M. Wood, J. J. Kuo, K. Imasato, G. J. Snyder. Improvement of Low‐Temperature zT in a Mg_3_Sb_2_–Mg_3_Bi_2_ Solid Solution via Mg‐Vapor Annealing. *Advanced Materials*, vol. 31, no. 35, pp. 1902337, 2019.
18. X. M. Shi, C. Sun, X. Y. Zhang, Z. W. Chen, S.Q. Lin, W. Li, Y. Z. Pei. Efficient Sc-doped Mg_3.05-x_Sc_x_SbBi thermoelectrics near room temperature. *Chemistry of Materials*, vol. 31, no. 21, pp. 8987-8994, 2019.
19. R. S. Zhai, Y. H. Wu, T.-J. Zhu, X.-B. Zhao. Tunable Optimum Temperature Range of High-Performance Zone Melted Bismuth-Telluride-Based Solid Solutions. *Crystal Growth & Design*, vol. 18, no. 8, pp. 4646-4652, 2018.
20. L. P. Hu, H. J. Wu, T. J. Zhu, C. G. Fu, J. Q. He, P. J. Ying, X. B. Zhao. Tuning multiscale microstructures to enhance thermoelectric performance of n‐type Bismuth‐Telluride‐based solid solutions. *Advanced Energy Materials*, vol. 5, no. 17, pp. 1500411, 2015.
21. W. S. Liu, K. C. Lukas, K. McEnaney, S. Lee, Q. Zhang, C. P. Opeil, G. Chen, Z. F. Ren. Studies on the Bi_2_Te_3_–Bi_2_Se_3_–Bi_2_S_3_ system for mid-temperature thermoelectric energy conversion. *Energy & Environmental Science*, vol. 6, no. 2, pp. 552-560, 2013.
22. R. Shu, Y. C. Zhou, Q. Wang, Z. J. Han, Y. B. Zhu, Y. Liu, Y. X. Chen, M. Gu, W. Xu, Y. Wang, W. Q. Zhang, L. Huang, W. S. Liu. Mg_3+ δ_Sb_x_Bi_2− x_Family: A Promising Substitute for the State‐of‐the‐Art n‐Type Thermoelectric Materials near Room Temperature. *Advanced Functional Materials*, vol. 29, no. 4, pp.1807235, 2019.
23. X. X. Chen, H. J. Wu, J. Cui, Y. Xiao, Y. Zhang, J. Q. He, Y. Chen, J. Cao, W. Cai, S. J. Pennycook, Z. H. Liu, L.-D. Zhao, J. H. Sui. Extraordinary thermoelectric performance in n-type manganese doped Mg_3_Sb_2_ Zintl: High band degeneracy, tuned carrier scattering mechanism and hierarchical microstructure. *Nano Energy*, vol. 52, pp. 246-255, 2018.
24. J. Mao, H. T. Zhu, Z. W. Ding, Z. H. Liu, G. A. Gamage, G. Chen, Z. F. Ren, “High thermoelectric cooling performance of n-type Mg3Bi2-based materials,” Science, vol. 365, no. 6452, pp. 495-498. 2019.
